# The clinical characteristics of non-cystic fibrosis bronchiectasis patients with positive serum tumor markers: a retrospective study

**DOI:** 10.1186/s12890-023-02816-7

**Published:** 2024-01-08

**Authors:** Xiaoyue Wang, Juan Wang, Siqi He, Jing Li, Xiaoting Chen, Tianyuan Ma, Lu Liu, Lei Zhang, Xiaoning Bu

**Affiliations:** 1 Department of Respiratory and Critical Care Medicine, Chuiyangliu Hospital affiliated to Tsinghua University, Beijing, China; 2 Department of Respiratory and Critical Care Medicine, Beijing Tiantan Hospital, Capital Medical University, Beijing, China; 3 Department of Respiratory and Critical Care Medicine, Beijing Chaoyang Hospital, Capital Medical University, Beijing, China; 4 Department of Respiratory and Critical Care Medicine, Fengtai rehabilitation hospital of Beijing Municipality (Tieying hospital), Beijing, China; 5 Department of Respiratory and Critical Care Medicine, Beijing Fangshan Liangxiang Hospital, Beijing, China

**Keywords:** Bronchiectasis, Serum tumor marker, Clinical characteristic, Indicators

## Abstract

**Background:**

Serum tumor markers (STM), extensively used for the diagnosis, monitoring and prognostic assessment of tumors, can be increased in some non-malignant lung diseases. To date, there is a paucity of studies regarding the clinical characteristics of non-cystic fibrosis bronchiectasis patients with positive STMs.

**Objective:**

To investigate the clinical characteristics and indicators of bronchiectasis with positive STMs.

**Methods:**

The clinical data of 377 bronchiectasis patients was retrospectively collected from January 2017 to December 2019 from Beijing Chaoyang Hospital. Patients were divided into the STM negative group, the single STM positive group and the ≥2 STMs positive group according to the number of the positive STMs. The clinical characteristics are described and compared separately. The multivariate logistic regression analysis model was used to investigate the indicators regarding positive STMs.

**Results:**

Patients in the ≥2 STMs positive group were older (*P* = 0.015), had higher mMRC scores (*P* < 0.001) and developed higher fever (*P* = 0.027). Additionally, these patients also had lower Albumin/Globulin Ratio (A/G), albumin (ALB), prealbumin (PAB) (*P* < 0.001, *P <* 0.001, *P* < 0.001, respectively) and higher CRP, ESR and Fbg (*P* < 0.001, *P* < 0.001 and *P* < 0.001, respectively). Age (OR 1.022, 95%CI 1.003–1.042; *P* = 0.026) and the number of affected lobes (OR 1.443, 95%CI 1.233–1.690; *P* < 0.001) were independently associated with one and ≥ 2 positive STMs in bronchiectasis patients.

**Conclusion:**

The ≥2 positive STMs are associated with a higher inflammation status and severer radiologic manifestations in bronchiectasis patients.

## Introduction

Non-cystic bronchiectasis (which was referred to bronchiectasis) is a chronic respiratory disease characterized by irreversible dilation of the bronchi and repeated purulent infections presenting with symptoms including cough, sputum production and hemoptysis [1, 2]. In China, the incidence of bronchiectasis was estimated to be 1.2% in residents over 40 years of old according to incomplete statistics and increased with age [3], which have placed a heavy burden on the health-care systems and the individuals [4, 5].

Serum tumor markers (STMs) are extensively used for the screening, early diagnosis, monitoring and prognostic assessment of tumor diseases [6]. However, in clinical practice, elevated STMs are common in patients without malignant tumors. Several previous studies have reported the association between non-malignant lung diseases and STMs [7–15]. Several case study reports have demonstrated that serum carbohydrate antigen199 (CA199) levels were significantly elevated in patients with allergic bronchopulmonary aspergillosis (ABPA), tuberculosis and bronchiectasis infected with *P. aeruginosa* and *H. influenzae* [7–11]. The elevated CA199 levels markedly decreased and even returned to normal after operations or medications [7–11]. A cross-sectional study demonstrated a strong association between carcinoembryonic antigen (CEA) levels and airway changes, mainly thickening bronchi and traction bronchiectasis on chest high-resolution computed tomography (HRCT) scans in patients with rheumatoid arthritis (RA) [13]. Another study demonstrated the level of serum squamous cell carcinoma antigen (SCC) increased in nonmalignant tumor disease including pneumonia, asthma, pulmonary tuberculosis, bronchiectasis, and interstitial pneumonia, with an average SCC-positive rate of 22.6% in bronchiectasis patients [14].

To our best of knowledge, few studies have described the elevated level of STMs in patients with bronchiectasis. Gu et al. found a rise of serum carbohydrate antigen 125 (CA125) levels in patients hospitalized with bronchiectasis and the abnormal CA125 levels declined after the anti-infection therapy [15]. However, this published literature only focused on CA125, other biomarkers such as CEA, carbohydrate antigen 724 (CA724), cytokeratin 19 fragment (CYFRA21-1*)* and neuron-specific enolase (NSE) were not evaluated. To date, there is still a paucity of studies regarding positive STMs as well as the independent indicators for STMs in bronchiectasis patients. The aim of this study was to investigate the clinical characteristics of bronchiectasis patients with positive STMs.

## Methods

### Study design and the population

This was a retrospective study conducted in Beijing Chaoyang Hospital affiliated to Capital Medical University between January 2017 and December 2019. Patients aged 18 years or older, with HRCT confirmed bronchiectasis with accordingly clinical symptoms and complete data for STMs (CYFRA21-1, CA199, SCC, NSE, CA125, CA724 and CEA) were included in the study. Exclusion criteria: 1) Patients diagnosed with or cannot be entirely excluded from malignant tumors 2) Patients with concomitant pulmonary disease e.g. interstitial lung disease, pleural disease, acute respiratory distress syndrome (ARDS), pulmonary embolism, cor pulmonale and end-stage lung disease awaiting lung transplantation 3) People with acute or chronic liver or renal insufficiency, heart failure and other significant medical conditions 4) Patients with acute infectious disease such as bacterial and viral pneumonia, urinary tract infection, and infectious gastroenteritis 5) Any medical condition that may interfere STMs e.g. skin diseases (such as eczema, psoriasis), gynecological diseases (such as endometriosis, ovarian cysts), hemorrhagic stroke, ischemic stroke, neurodegenerative diseases (such as Alzheimer’s disease and Parkinson’s disease), collagen disease and pancreatitis. The Institutional Review Board of Beijing Chaoyang Hospital affiliated to Capital Medical University approved the study (2016-k-017) and written informed consent was obtained from all patients.

### Measurements

Data was extracted from electronic medical records, including participant demographics (age, sex, body mass index (BMI) and smoke status), the etiology of bronchiectasis (idiopathic, post-infective, post-tuberculosis and others), presence of comorbidities (cerebrovascular disease, coronary heart disease, hypertension, diabetes, chronic obstructive pulmonary disease (COPD) and asthma), clinical symptoms (cough, sputum, fever, mMRC dyspnea score and hemoptysis), disease duration, laboratory results (pH, PaO_2_/FiO_2_ Ratio, PaCO_2_, white blood cell (WBC), neutrophil (NEU), CRP, procalcitonin (PCT), ESR, fibrinogen (Fbg), D-dimer, albumin (ALB), prealbumin (PAB), globulin (GLB), albumin/globulin Ratio (A/G), STMs including CYFRA21-1, CA199, SCC, NSE, CA125, CA724 and CEA. STMs were measured by electrochemiluminescent immunoassay. Blood and sputum samples were collected on admission. The radiological manifestations included the number of affected lobes, unilateral/bilateral ratio and involvement of lobes on chest HRCT imaging. Additionally, the length of hospitalization was also recorded.

### Procedures

#### The definition of positive STMs

The values of STM exceed the normal range were STM positive and none were STM negative. The normal range for these tumor markers were: CYFRA21-1<2.08 ng/ml, CA199<37 U/ml, SCC<1.5 ng/ml, NSE<16.3 ng/ml, CA125<30.2 U/ml, CA724<8.2 U/ml and CEA<5 ng/ml.

#### The definition of chest HRCT confirmed bronchiectasis

The chest HRCT confirmed bronchiectasis was defined as abnormal widening and thickening of its airway wall; an irregular wall and lack of tapering, and/or visualization of bronchi in the periphery of the lung [16].

#### The definition of idiopathic bronchiectasis

The idiopathic bronchiectasis was defined as bronchiectasis with no identifiable casual, initiating factor after an exhaustive anamnesis and a minimum battery of aetiological tests [17].

#### The definition of fever

Fever was defined as having body temperature ≥ 37.3 °C [18].

#### The definition of disease duration

Disease duration was defined as the time since the diagnosis of the disease according to radiological manifestations and clinical symptoms.

Patients meeting the inclusion criteria were divided into the ≥2 positive STMs group, the single STM positive group, and the STM negative group based on the number of positive STMs. Those patients with positive STMs were also divided into seven groups according to different subtypes of STMs.

### Statistical analysis

The data were double checked and then collected using the Microsoft 26.0. Statistical analysis was analyzed using SPSS version 25.0 and visualized using Graphpad Prism Version 8.0. Normally distributed continuous variables were expressed as the mean ± standard deviation and non-normally distributed continuous variables were summarized as medians with interquartile range (IQR). Differences between the two groups were tested with a two-independent samples t-test and Mann–Whitney U-test for the comparison of each positive STM patients with negative STM patients, respectively. Continuous data were compared using the nonparametric Kruskal-Wallis test for the comparison between the ≥2 positive STMs group, the single STM positive group and the STM negative group. Categorical variables were summarized as percentage and were analyzed using a Chi-squared test or Fisher’s exact probability method. Bonferroni method correction was used for multiple comparisons. Multivariate regression analysis was conducted using a forward stepwise selection procedure and was used to analyze the independent indicators for positive STM in patients with bronchiectasis. The strength of the association was expressed as odd ratio (OR) and 95% confidential intervals (95% CI). Only factors with significant *P* values in the univariate analysis were included in the multivariate analysis. Multicollinearity was tested using a variance inflation factor (VIF). The interdependent variables (VIF > 5) were removed. Therefore, the variables such as age, fever, mMRC, disease duration, CRP, PCT, ESR, Fbg, A/G, PAB and the number of affected lobes were included in the multivariate analysis of the one and ≥ 2 STMs positive group versus the negative STM group. The variables such as age, sex, BMI, COPD, fever, mMRC, WBC, NEU, CRP, ESR, Fbg, D-Dimer, PAB, A/G, organism identified and the number of affected lobes were included in the multivariate analysis of the ≥2 STMs positive group versus the one STM positive group versus the negative positive group. A two-sided *P* value < 0.05 was considered statistically significant.

## Results

### Patient characteristics

**Fig. 1:**
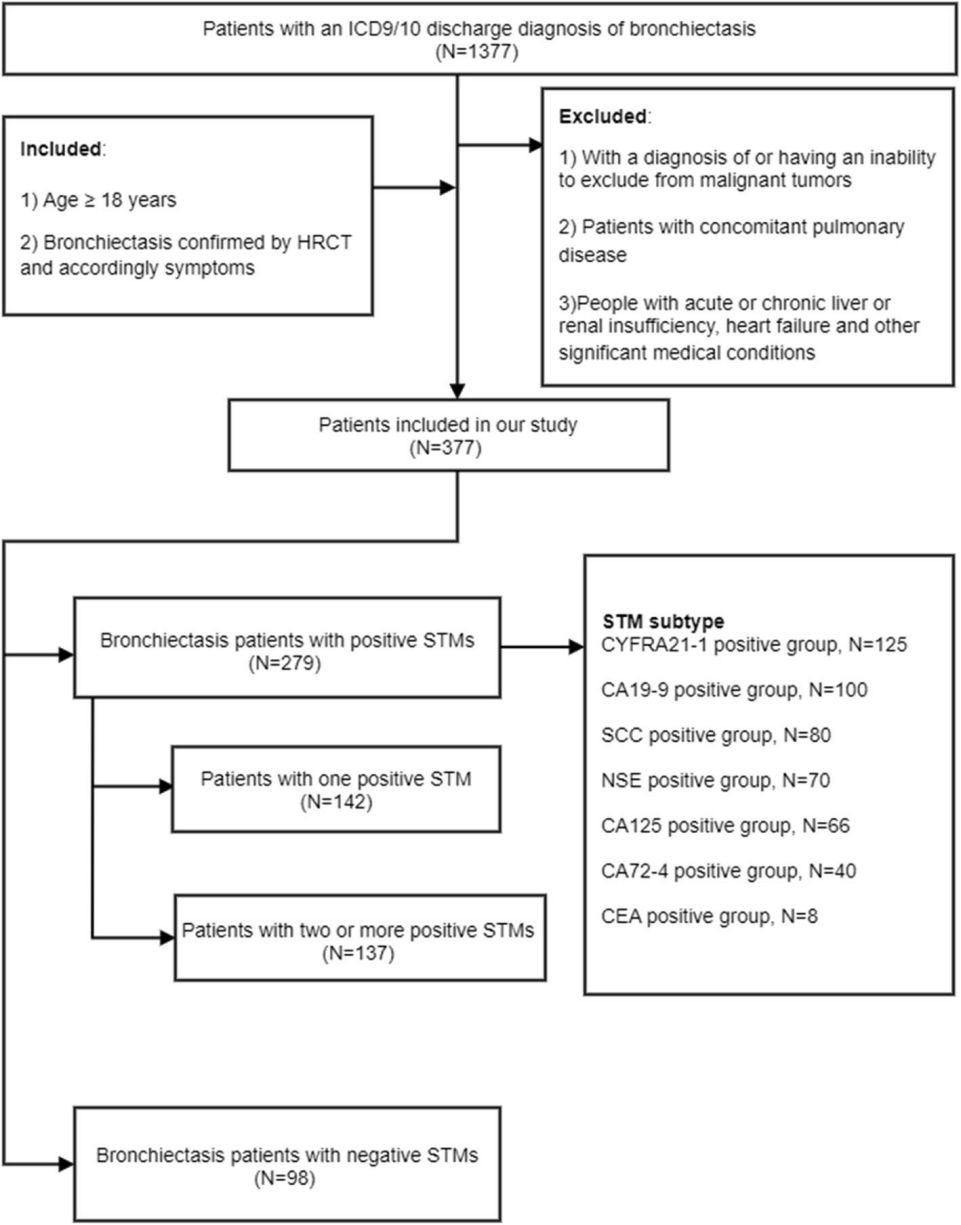
Flowchart of the study population

Of the 1377 patients with an international Classification of Diseases, 9th or 10th Revision, Clinical Modification codes (ICD-9-CM or ICD-10-CM) ICD9/10 discharge diagnosis of bronchiectasis between January 2017 and December 2019, 377 patients were included in our study according to the inclusion and exclusion criteria. The overall study flow chart was shown in Fig. 1. Among the 377 patients, their median age was 62 (54,67) years, with a predominance of females (67.1%). Almost three fourth (*n* = 279) of the patients had positive STM results, with the remaining (*n* = 98) having negative STM results. Of those positive STM results, positive CYFRA21-1 results (*n* = 125) predominated in patients with bronchiectasis, followed by positive CA199 results (*n* = 100), positive SCC results (*n* = 80), positive NSE (*n* = 70), positive CA125 (*n* = 66), positive CA72–4 results (*n* = 40) and positive CEA results (n = 8).

### Comparison of patients with ≥2 positive STMs vs patients with one positive STM vs patients with negative STMs

**Fig. 2:**
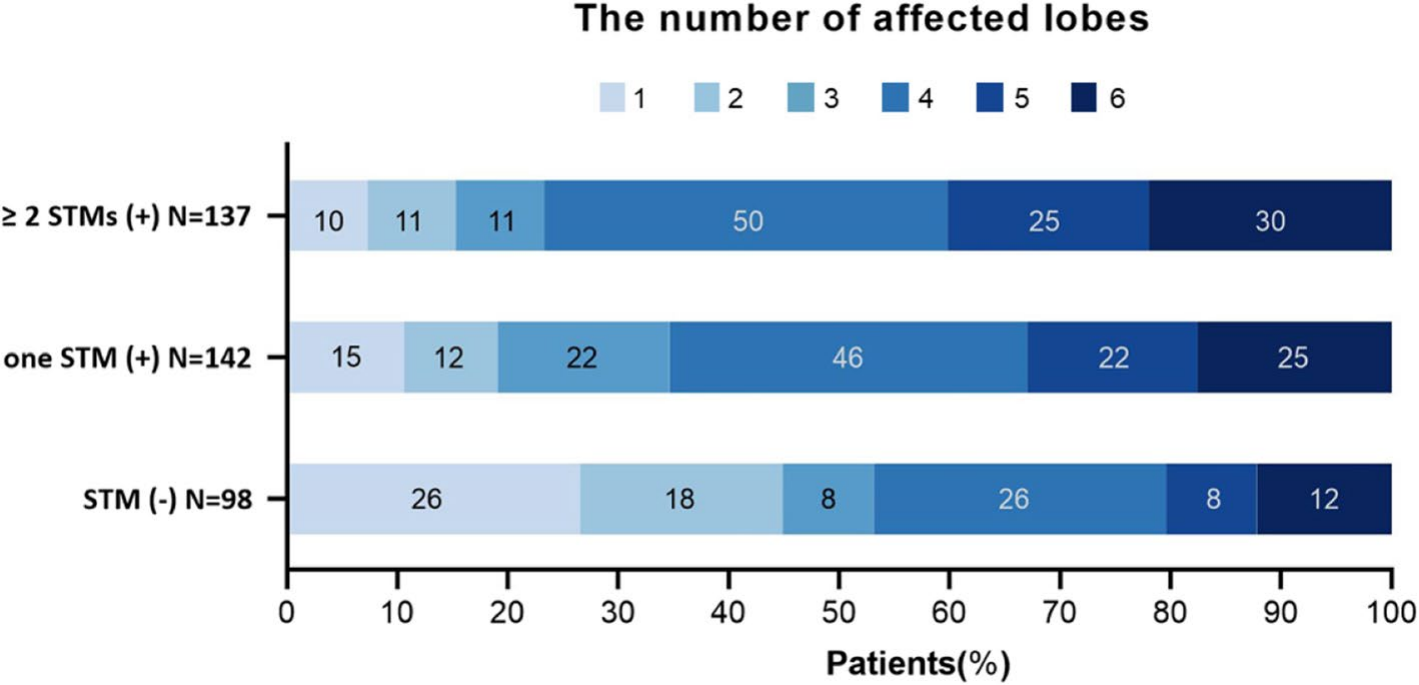
The number of affected lobes in STM negative, one STM positive and ≥ 2 positive STMs group

The positive STM group was categorized into two groups according to the number of positive STMs: the one STM positive group (*n* = 142) and the ≥2 positive STMs group (*n* = 137). The demographic and clinical characteristics were summarized in the Table 1. The median age was significantly higher in patients with ≥2 positive STMs than with negative STM (*P* = 0.015), with 19.7% patients over 70 years of age. Patients with ≥2 positive STMs also had lower BMI than the other two groups (*P* < 0.001). The ≥2 positive STMs group had a higher proportion of mMRC dyspnea score and fever than the one positive STM group and the negative STM group (*P* < 0.001 and *P* = 0.027, respectively). The ≥2 positive STMs group also had lower ALB, A/G, PAB and GLB than the other two groups (*P* < 0.001, *P* < 0.001, *P* < 0.001 and *P* = 0.004, respectively). Patients with ≥2 positive STMs also had higher CRP, ESR, Fbg, D-dimer than those with one positive STM and those with negative STMs (*P <* 0.001, *P <* 0.001, *P* < 0.001, *P* = 0.001, respectively). The Fig. 2 represents the number of affected lobes in different groups. Additionally, the one positive STM group and the ≥2 positive STMs group had a higher number of affected lobes and more bilateral lobe involvement (*P* = 0.001 and *P* = 0.001, respectively). Patients with bronchiectasis were further categorized according to the subgroup of STMs. Table 2 provides an overview of the demographic and clinical features of bronchiectasis patients with each positive STM.

**Table 1:** Clinical characteristics according to the number of positive STMs in NCFB patients

Variables	≥ 2 STMs positive (*n* = 137)	one STM positive (n = 142)	STM negative (n = 98)	*P Value*
Demographics				
Age, y	62 (55,69)	62 (54,68)	59 (47,65)	0.015^a^
Sex, F, n (%)	78 (56.9)	105 (73.9)	70 (71.4)	0.006^c^
BMI, kg/cm^2^	20.0 (18.1,23.6)	22.8 (20.2,25.5)	22.5 (19.8,24.5)	<0.001^a,c^
Smoke status, n (%)				0.466
Never	108 (78.8)	122 (85.9)	79 (80.6)	
Past	19 (13.9)	11 (7.7)	10 (10.2)	
Current	10 (7.3)	9 (6.3)	9 (9.2)	
Etiology, n (%)				0.225
Idiopathic	67 (48.9)	88 (62.0)	59 (60.2)	
Post-infective	24 (17.5)	23 (16.2)	15 (15.3)	
Post-tuberculosis	30 (21.9)	16 (11.3)	13 (13.3)	
Others	16 (11.7)	15 (10.6)	11 (11.2)	
Comorbidities, n (%)				
Cerebrovascular disease	6 (4.4)	7 (4.9)	1 (1.0)	0.254
Coronary heart disease	9 (6.6)	9 (6.3)	5 (5.1)	0.888
Hypertension	29 (21.2)	40 (28.2)	21 (21.4)	0.315
Diabetes	15 (10.9)	10 (7.0)	8 (8.2)	0.500
COPD	8 (5.8)	2 (1.4)	1 (1.0)	0.039^a^
Asthma	9 (6.6)	9 (6.3)	10 (10.2)	0.475
Clinical symptoms, n (%)				
Cough	137 (100)	140 (98.6)	95 (96.9)	0.129
Sputum	131 (95.6)	129 (90.8)	86 (87.8)	0.085
Fever	55 (40.1)	38 (26.8)	20 (20.4)	0.027^a, c^
mMRC				<0.001^a, c^
≤ 2	59 (43.1)	88 (62.0)	65 (66.3)	
> 2	78 (56.9)	54 (38.0)	33 (33.7)	
Hemoptysis	36 (26.3)	50 (35.2)	40 (40.8)	0.057
Disease duration, y	2 (1,15)	2 (0,15)	1 (0,10)	0.057
Laboratory				
WBC (× 10^9^ mL − 1)	6.80 (5.62,9.07)	6.22 (4.98,7.35)	6.19 (5.12,7.56)	0.003^c^
NEU (×10^9^ mL − 1)	4.30 (3.24,6.48)	3.68 (2.66,4.67)	3.79 (2.88,4.85)	<0.001^a,c^
CRP, mg/ L	13.8 (5.3,6.6)	5.4 (2.9,13.8)	5.1 (2.5,11.6)	<0.001^a,c^
PCT ≥ 0.5, ng/L	8 (5.8)	1 (0.7)	3 (3.1)	0.278
ESR, mm/h	26 (11,48)	12 (5,26)	12 (5,20)	<0.001^a,c^
Fbg, g/L	3.87 (3.20,4.83)	3.16 (2.71,3.75)	2.93 (2.56,3.59)	<0.001^a,c^
D-Dimer, ng/mL	530 (310,1263)	370 (240,586)	340 (225,663)	0.001^a,c^
ALB, g/L	37.6 (34.4,39.5)	39.3 (37.0,41.2)	38.6 (37.0,41.1)	<0.001^a,c^
PAB, mg/dl	15 (10,21)	19 (15,24)	19 (14,24)	<0.001^a,c^
GLB, g/L	29.5 (26.8,34.9)	28.3 (25.8,31.3)	27.9 (25.5,30.9)	0.004^a,c^
A/G	1.3 (1.0,1.5)	1.4 (1.2,1.5)	1.4 (1.3,1.6)	<0.001^a,c^
Arterial blood gas analysis				
pH	7.43(7.41,7.45)	7.42(7.40,7.45)	7.42(7.41,7.44)	0.550
PaO2/FiO2 Ratio	352(311,399)	367(325,419)	364(329,430)	0.134
PaCO2, mmHg	43(40,47)	42(39,47)	42(39,45)	0.434
Microbiology				
Organism identified	53 (38.7)	38 (26.8)	24 (24.5)	< 0.031^a, c^
*P. aeruginosa*	31 (22.6)	25 (17.6)	13 (13.3)	0.181
Others	22 (16.1)	13 (9.2)	11 (11.2)	0.200
Radiological				
The number of affected lobes	4 (4,5)	4 (3,5)	3 (1,4)	0.001^a, b,^
Unilateral/Bilateral	19/118	27/115	35/63	0.001^a, b^
Involvement of lobes				
Left upper lobe	75 (54.7)	66 (46.5)	37 (37.8)	0.036^a^
Lingula	108 (78.8)	102 (71.8)	54 (55.1)	<0.001^a, b^
Left lower lobe	93 (67.9)	97 (68.3)	54 (55.1)	0.068
Right upper lobe	87 (63.5)	85 (59.9)	50 (51.0)	0.152
Right middle lobe	101 (73.7)	103 (72.5)	53 (54.1)	0.002^a, b^
Right lower lobe	106 (77.4)	96 (67.6)	54 (55.1)	0.001^a, b^
Length of hospital, d	11 (9,14)	11 (8,13)	11 (8,13)	0.348

Definition of abbreviations: *BMI* body mass index, *COPD* chronic obstructive pulmonary disease, *mMRC* modified British medical research council, *WBC* white blood cell, *NEU* neutrophil, *CRP* C-reactive protein, *PCT* procalcitonin, *ESR* erythrocyte sedimentation rate, *Fbg* fibrinogen, *ALB* albumin, *PAB* prealbumin, *A/G* Albumin/Globulin Ratio, *PaO2* partial pressure of oxygen, *PaCO2* partial pressure of carbon dioxide; Data are presented as median (IQR) for continuous variables and number (percentage) for categorized variables. *p*-value< 0.05

a ≥ 2 STMs positive vs STM negative, b one STM positive vs STM negative, c ≥ 2 STMs positive vs one STM positive

**Table 2:** Clinical characteristics according to the presence of each positive STM in NCFB patients

Variables	CYFRA21-1 (+) n = 125	CA199 (+) n = 100	SCC (+) *n* = 80	NSE (+) *n* = 70	CA125 (+) *n* = 66	CA724 (+) *n* = 40	CEA (+) n = 8
Demographics							
Age, y	63 (56,70)^*^	62 (55,69)^*^	63 (55,67)^*^	62 (54,68)^*^	64 (56,71)^*^	62 (51,67)	55 (44,62)
Sex, F, n (%)	78 (62.4)	72 (72.0)	38 (47.5)^*^	45 (64.3)	37 (56.1)^*^	24 (60.0)	5 (62.5)
BMI, kg/cm^2^	22.1 (19.1,25.3)	20.4 (18.2,23.7)^*^	20.0 (18.0,23.8)^*^	22.5 (19.6,24.2)	19.6 (17.3,22.9)^*^	20.4 (18.0,23.5)^*^	22.9 (21.2,27.2)
Clinical symptoms, n (%)							
Fever	34 (27.2)	39 (34.8)^*^	31 (38.8) ^*^	26 (37.1)^*^	25 (37.9) ^*^	18 (45.0)^*^	1 (0.9)
mMR C > 2	64 (51.2)^*^	54 (54.0)^*^	49 (61.3)^*^	31 (44.3)	40 (60.6)^*^	23 (57.5)^*^	7 (87.5)^*^
Disease duration, y	2 (0,10)	4 (0,22)^*^	2 (0,14)	5 (0,13)	5 (0,20)^*^	4 (0,17)	0 (0,25)
Laboratory							
WBC (×10^9^ mL − 1)	6.44 (5.08,8.15)	6.71 (5.55,8.40)	7.04 (5.52,9.19)	6.38 (5.27,8.75)	7.28 (5.89,9.68)^*^	7.48 (6.46,10.46)^*^	7.38(6.07,11.5)
NEU (×10^9^ mL − 1)	3.93 (2.76,5.33)	4.21 (3.10,5.69)	4.36 (2.99,6.46)^*^	3.92 (2.93,6.27)	5.12 (3.72,7.58)^*^	4.98 (3.91,7.29)^*^	4.35(3.28,10.00)
CRP, mg/ L	6.8 (2.9,19.6)	9.7 (4.1,38.9)^*^	15.2 (5.7,74.7)^*^	10.0 (3.0,31.7) ^*^	25.6 (6.8,79.0)^*^	25.9 (6.2,83.6)^*^	2.7(2.4,12.5)
ESR, mm/h	14 (5,34)	24 (12,41)^*^	24 (10,48)^*^	19 (9,37)^*^	34 (20,54)^*^	34 (11,49)^*^	6 (3,26)
Fbg, g/L	3.41 (2.64,4.02)^*^	3.71 (3.05,4.61)^*^	3.79 (3.20,4.82)^*^	3.45 (2.91,4.61)^*^	4.52 (3.57,5.48)^*^	4.09 (3.22,5.40)^*^	2.81 (2.17,3.68)
D-Dimer, ng/mL	386 (248,720)	416 (282,793)	518 (295,1047) ^*^	508 (298,959)^*^	899 (499,1706)^*^	735 (324,1552) ^*^	222 (190,404)
ALB, g/L	38.9 (36.3,41.0)	38.0 (35.8,39.9)	36.6(33.7,39.6)^*^	38.2 (35.9,39.8)	36.2 (32.8,38.6)^*^	37.4 (32.8,41.4)^*^	40.2 (38.0,43.11)
PAB, ng/dL	19 (14,24)	15 (11,22)^*^	14 (9,20)^*^	18 (13,23)	10 (7,16)^*^	15 (7,20)^*^	29 (23,30)^*^
GLB, g/L	28.0 (25.5,32.4)	29.5 (26.2,33.1)^*^	29.6 (26.7,35.1)^*^	28.6 (26.5,33.4)	32.1 (27.5,38.3)^*^	32.3 (26.5,38.3)^*^	29.4 (26.4,31.1)
A/G	1.4 (1.2,1.5)	1.3 (1.1,1.5)^*^	1.3 (1.0,1.5)^*^	1.4 (1.1,1.5)^*^	1.2 (0.9,1.4)^*^	1.2 (0.9,1.5)^*^	1.4 (1.3,1.5)
Microbiology, n (%)							
Organism identified	41 (32.8)	45 (45.0)^*^	30 (37.5)	17 (24.3)	27 (40.9)^*^	16 (40.0)	3 (37.5)
*P. aeruginosa*	21 (16.8)	28 (28.0)^*^	22 (27.5)^*^	12 (17.1)	17 (25.8)^*^	7 (17.5)	1 (12.5)
Others	20 (16)	17 (17)	8 (10)	5 (7.1)	10 (15.2)	9 (22.5)	2 (25.0)
Radiological							
The number of affected lobes	4 (3,5)^*^	4 (4,5)^*^	4 (4,6)^*^	4 (3,5)^*^	5 (4,5)^*^	4 (3,5)^*^	4 (2,4)
Unilateral/Bilateral, n.	21/104^*^	14/86^*^	7/73^*^	14/56^*^	3/63^*^	6/34^*^	1/7
Involvement of lobes, n (%)							
Left upper lobe	49 (39.2)	46 (46.0)^*^	43 (53.8)^*^	26 (37.1)	37 (56.1)^*^	16 (40.0)	1 (12.5)
Lingula	96 (76.8)^*^	85 (85.0)^*^	70 (87.5)^*^	45 (64.3)	64 (97.0)^*^	31 (77.5)^*^	5 (62.5)
Left lower lobe	95 (76.0)	89 (89.0)^*^	67 (83.8)^*^	54 (77.1)	61 (92.4)^*^	29 (72.5)	7 (87.5)
Right upper lobe	71 (56.8)	65 (65.0)^*^	52 (65.0)^*^	37 (52.9)	51 (77.3)^*^	25 (62.5)	2 (25.0)
Right middle lobe	95 (76.0)^*^	77 (77.0)^*^	63 (78.8)^*^	44 (62.9)	60 (90.9)^*^	29 (72.5)^*^	6 (75.0)
Right lower lobe	93 (74.4)^*^	77 (77.0)^*^	70 (87.5)^*^	49(70.0)	58 (87.9)^*^	33 (82.5)^*^	7 (87.5)
Length of hospital, d	11 (9,13)	11 (8,14)	11 (8,14)	11 (8,14)	13 (10,16)^*^	14 (9,18)^*^	12 (7,16)

Definition of abbreviations: *BMI* body mass index, *COPD* chronic obstructive pulmonary disease, *mMRC* modified British medical research council, *WBC* white blood cell, *NEU* neutrophil, *CRP* C-reactive protein, *PCT* procalcitonin, *ESR* erythrocyte sedimentation rate, *Fbg* fibrinogen, *ALB* albumin, *PAB* prealbumin, *A/G* Albumin/Globulin Ratio, *PaO2* partial pressure of oxygen, *PaCO2*, partial pressure of carbon dioxide; Data are presented as median (IQR) for continuous variables and number (percentage) for categorized variables. * *p*-value< 0.05

### Comparison of patients with each positive STM vs patients with negative STM

The clinical characteristics of patients with each positive STM were summarized in Table 2. Patients with each positive STM were older except for CA724 and CEA. Patients with positive CA199, NSE, CA125 and CA724 had lower body weight than those with negative STM. We also observed that patients with positive CA199, SCC, CA125, CA724 and CEA had poorer nutritional status (evaluated by ALB and PAB). CRP and ESR levels were significantly higher in patients with positive CA199, SCC, NSE, CA125 and CA724. In terms of the radiological manifestations, a higher number of affected lobes and a higher proportion of bilateral involvement were observed in patients with each positive STM except for CEA.

### Multivariate analysis

**Fig. 3:**
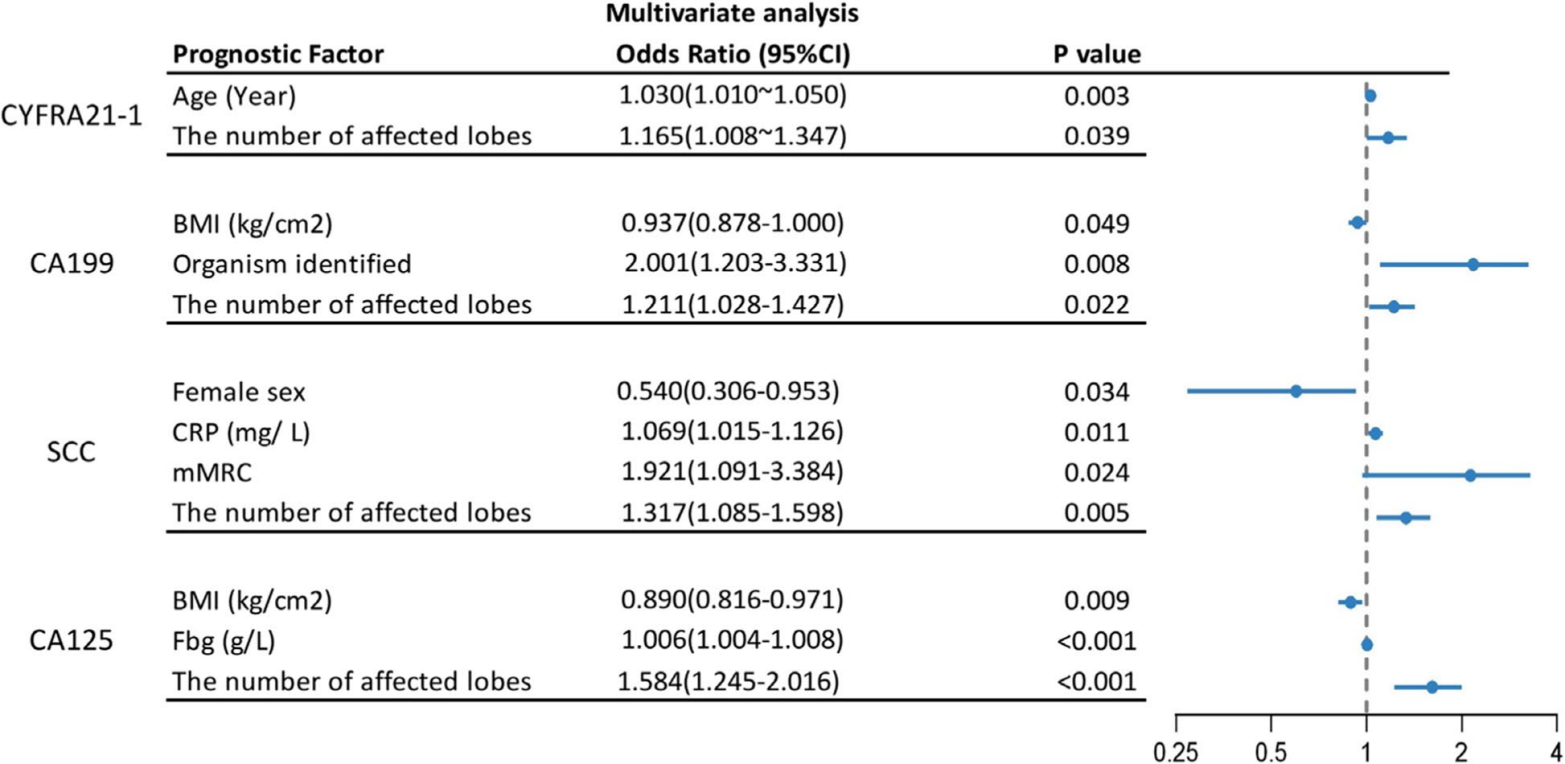
The indicators for each positive STM in bronchiectasis patients. Definition of abbreviations: BMI, body mass index; mMRC modified British medical research council

Multivariate logistic regression analysis showed that age (OR 1.022, 95%CI 1.003–1.042; *P* = 0.026) and the number of affected lobes (OR 1.443, 95%CI 1.233–1.690; *P* < 0.001) were independently associated with one and two or more positive STMs in bronchiectasis patients (Table 3). ESR (OR 1.026, 95%CI 1.011–1.041; *P* = 0.001), BMI (OR 0.870, 95%CI 0.806–0.939; *P* < 0.001) and female sex (OR 0.415, 95%CI 0.234–0.735; *P* = 0.003) significantly differentiated between the ≥2 positive STMs and the one positive STM group. ESR (OR 1.028, 95%CI 1.010–1.045; *P* = 0.002), female sex (OR 0.484, 95%CI 0.258–0.907; *P* = 0.024) and the number of affected lobes (OR 1.316, 95%CI 1.082–1.602; *P* = 0.006) were independently associated with ≥2 positive STMs in the ≥2 positive STMs versus the STM negative comparison (Table 4). The number of affected lobes (OR 1.395, 95%CI 1.159–1.679; *P* < 0.001) was independently associated with one positive STM in bronchiectasis patients (Table 4). Results from the multivariate analysis for each STM were shown in Fig. 3. Age (OR 1.030, 95%CI 1.010–1.050; *P* = 0.003) and the number of affected lobes (OR 1.165, 95%CI 1.008–1.347; *P* = 0.039) were independent indicators for positive CYFRA21-1. BMI (OR 0.937, 95%CI 0.878–1.000; *P* = 0.049), organism identified (OR 2.001, 95%CI 1.203–3.331; *P* = 0.008) and the number of affected lobes (OR 1.211, 95%CI 1.028–1.427; *P* = 0.022) were independent indicators for positive CA199. CRP (OR 1.069, 95%CI 1.015–1.126; *P* = 0.011), mMRC (OR 1.921, 95%CI 1.091–3.384; *P* = 0.024), the number of affected lobes (OR 1.317, 95%CI 1.085–1.598; *P* = 0.005) and female sex (OR 0.540, 95%CI 0.306–0.953; *P* = 0.034) were independent indicators for positive SCC. Fbg (OR 1.006, 95%CI 1.004–1.008; *P* < 0.001), BMI (OR 0.890, 95%CI 0.816–0.971; *P* = 0.009) and the number of affected lobes (OR 1.584, 95%CI 1.245–2.016; *P* < 0.001) were independent indicators for positive CA125. There were no independent indicators for positive NSE and CA724.

**Table 3:** Parameter estimates, odds ratios for the final binary logistic regression analysis

Variables	≥ 2 and one positive STMs versus STM negative				*P* value
	Estimate (SE)	Odds ratio	95% Wald CL for odds ratio		
			Lower	Upper	
Intercept	−1.288(0.684)	NA	NA	NA	0.060
Age, y	0.022(0.010)	1.022	1.003	1.042	0.026
A/G	−0.174(0.158)	0.840	0.617	1.145	0.271
The number of affected lobes	0.367(0.080)	1.443	1.233	1.690	< 0.001

Definition of abbreviations: *A/G* Albumin/Globulin Ratio

**Table 4:** Parameter estimates, odds ratios for the final multinomial logistic regression analysis

Variables	≥ 2 positive STMs versus one STM positive				*P* value	≥ 2 positive STMs versus STM negative				*P* value	One STM positive versus STM negative				*P* value
	Estimate (SE)	Odds ratio	95% Wald CL for odds ratio			Estimate (SE)	Odds ratio	95% Wald CL for odds ratio			Estimate (SE)	Odds ratio	95% Wald CL for odds ratio		
			Lower	Upper				Lower	Upper				Lower	Upper	
Intercept	3.245(1.030)	NA	NA	NA	0.002	1.033(1.118)	NA	NA	NA	0.355	−2.212(1.050)	NA	NA	NA	0.035
Female	−0.879(0.292)	0.415	0.234	0.735	0.003	−0.727(0.321)	0.484	0.258	0.907	0.024	0.152(0.322)	1.165	0.620	2.187	0.635
BMI, kg/cm2	−0.139(0.039)	0.870	0.806	0.939	< 0.001	−0.084(0.044)	0.919	0.844	1.001	0.054	0.055(0.040)	1.056	0.977	1.142	0.168
ESR, mm/h	0.025(0.007)	1.026	1.011	1.041	0.001	0.027(0.009)	1.028	1.010	1.045	0.002	0.002(0.009)	1.002	0.984	1.020	0.822
The number of affected lobes	−0.058(0.093)	0.944	0.786	1.133	0.535	0.275(0.100)	1.316	1.082	1.602	0.006	0.333(0.095)	1.395	1.159	1.679	< 0.001

Definition of abbreviations: *BMI* body mass index, *ESR* erythrocyte sedimentation rate * *p*-value< 0.05

## Discussion

Positive STMs frequently occur in patients with bronchiectasis in clinical practice. A previous study has demonstrated that the rise in CA125 levels in patients with bronchiectasis declined after anti-inflammation therapy, indicating that CA125 might be associated with inflammatory markers [15]. However, studies on the association between multiple STMs and bronchiectasis are scarce. To our best of knowledge, this is the first clinical study that evaluates the indicators for the multiple positive STMs in patients with bronchiectasis. This present retrospective study found the ≥2 positive STMs group was higher in the age, mMRC, fever, the number of affected lobes, the inflammatory markers including ESR, CRP and Fbg compared with the negative STM group. Age and the number of affected lobes were independently associated with one and ≥ 2 positive STMs in bronchiectasis patients. The number of affected lobes was independently associated with one positive STM in bronchiectasis patients. Female sex, ESR and the number of affected lobes was independently associated with ≥2 positive STMs in bronchiectasis patients.

In our study, we observed that patients with CYFRA21-1, CA199, SCC, NSE and CA125 were older. Age was also an independent indicator for positive CYFRA21-1 in bronchiectasis patients. Previous studies found that CA199、CYFRA21-1 increased with age in healthy people^32–34^, which corroborates our results. Around 30% patients with bronchiectasis had positive CA199. The identified organism was independently associated with positive CA199. Previous studies have reported the markedly elevated CA199 in bronchiectasis patients, particularly in those infected with *P. aeruginosa* and *H. influenzae* [7–11], which might support our finding indirectly.

CRP and ESR, as markers of inflammation, are widely used for estimation of inflammatory states in clinical practice [19, 20]. Previous studies indicated ESR was more beneficial for chronic inflammatory diseases while CRP had higher predictive value for acute inflammation conditions [19, 20]. In this study, CRP and ESR were significantly higher in patients with ≥2 positive STMs in comparison with those with one positive STM or negative STMs. We also found a statistically significant elevation of CRP and ESR between each positive STM group and the STM negative group except for the CYFRA21-1 and the CEA positive group. Besides this, ESR was an independent indicator for ≥2 STMs positive STMs in bronchiectasis patients. CRP was also an independent indicator for positive SCC. Gu et al. discovered that a rise of CA125 in bronchiectasis patients declined after anti-inflammation therapy and CRP and TNF-α were independent indicators for the elevated CA125 in bronchiectasis patients [15], indicating the potential relationship between inflammation and elevated STMs. The potential reason might be that during the ongoing infection process, the production of inflammation may be directly or indirectly involved in regulating the expression of STM. However, the specific mechanism remains to be further studied. A study from Spain demonstrated CRP levels in stable bronchiectasis was a predictive factor for future severe exacerbations of bronchiectasis patients, with higher CRP levels associated with an increased risk of severe exacerbations [21, 22]. Elevated ESR was also reported to be an independent predictive factor for mortality [22]. Therefore, it is hypothesized that positive STMs could exhibit poorer prognosis in bronchiectasis patients. Future studies should look into the clinical outcomes of bronchiectasis patients with positive STMs.

BMI, serum ALB and PAB were commonly considered as markers for reflecting nutritional status. A previous study demonstrated that a substantial number of patients with bronchiectasis presented with low BMI in comparison to healthy people [23, 24]. Bronchiectasis patients also presented with lower BMI and ALB compared with those with other respiratory disorders like COPD, neuromuscular disease, restrictive disorders and mixed respiratory failure [25]. In this study, the ≥2 positive STM group had lower BMI, A/G and PAB than the other two groups. We also observed that patients with positive CA199, SCC, CA125, CA724 and CEA had lower ALB and PAB, implying that these patients have poor nutritional status. BMI was an independent indicator for serum positive CA199 and CA125. Previous studies have proven that nutritional status might affect the prognosis of bronchiectasis patients [26–28]. Researchers discovered a strong correlation between BMI, ALB, PAB and the bronchiectasis severity index, FACED (F: forced expiratory volume in 1 s [FEV1]; A: age; C: chronic colonization by *P. aeruginosa* [PA], E: radiological extension [number of pulmonary lobes affected], and D: dyspnea) and clinical symptoms in bronchiectasis patients [26, 29]. Furthermore, Li et al. found an inverse correlation between PAB, ALB, BMI and modified Reiff score and the number of involved lobes [29]. Zhao et al. also found that bronchiectasis patients with higher nutritional risk had significantly more acute exacerbations within the one-year follow up and required longer courses of antibiotic therapy [27]. BMI serves as an independent indicator for mortality in patients with bronchiectasis [28]. Therefore, it is reasonable to hypothesize that positive STMs might affect the prognosis of bronchiectasis patients by the poor nutritional status. As this study lack follow-up data, future prospective studies are needed to investigate the influence of positive STMs on the prognosis of bronchiectasis patients. Nutritional support and necessary nutritional interventions might be required for bronchiectasis patients with positive STMs to improve the nutritional status of this population and to improve the prognosis of bronchiectasis patients.

In this study, we found a strong correlation between the number of affected lobe involvement and the positive STM. The number of affected lobe involvement was also independently associated with positive CA199, CYFRA21-1, SCC and CA125. A previous study found the high levels of CEA was strongly associated with airway changes in RA, of which bronchi thickening and traction bronchiectasis were the two most common CT findings [13], which might confirm our findings in another way. Furthermore, we found that there was a significantly rise of bilateral lung involvement in bronchiectasis patients with one positive STM or ≥ 2 positive STMs, independently of the number of STMs. The potential explanation was that bilateral lung involvement and the widespread affected lobe involvement functioned more in the inflammation of bronchial tree and damage to the lung parenchyma, which was believed to contribute to the positive STM in bronchiectasis patients. A higher proportion of bilateral lung involvement was observed in bronchiectasis patients with lung function impairment in comparison with those with normal ventilation [30], which might indirectly reflect the poor prognosis of bronchiectasis patients with positive STMs. Another study from Korea demonstrated that BMI was an independent predictor for the progression of radiologic manifestations in bronchiectasis patients [31], which might partly explain the reason for the more severe radiologic manifestations in bronchiectasis patients with positive STMs.

In this study, female sex was independently associated with two or more STMs in bronchiectasis patients, with a less proportion of females in the ≥2 positive STMs group, however, the complete mechanism was not understood. Patients with positive STMs were more likely to present with fever and had higher mMRC scores, which was possibly caused by the higher inflammation status.

This is the first study exploring the association between multiple STMs and bronchiectasis patients. However, this study also had several limitations. Firstly, this study was a single-center retrospective observational study in a territory referral hospital, thereby possibly contributing to selection bias. Demographic, clinical, laboratory, microbiologic and radiology data were collected from electronic medical records. Secondly, this study was a retrospective one. The prognosis of bronchiectasis patients with positive STMs cannot be evaluated as these patients were not followed up. In addition, there was a lack of a healthy control group with elevated STMs, thus, the incidence of elevated STMs were not compatible between bronchiectasis patients and normal people. Future multicenter prospective studies with larger samples are needed to look into the indicators for elevated STMs in bronchiectasis patients as well as the relationship and the underlying mechanisms between elevated STMs and inflammatory markers. Furthermore, positive STMs may indicate a more serious condition, and warrant medical attention. Further studies should focus on elucidating whether the positive STMs observed in bronchiectasis can translate into adverse outcomes after exclusion of malignant tumors.

In summary, bronchiectasis patients with positive STMs were older, had longer disease duration, more clinical symptoms, poorer nutritional status, higher levels of inflammation markers and higher numbers of lobe involvement compared with those without STMs, indicating that the positive STM is associated with a higher inflammation status and severer radiologic manifestations in bronchiectasis patients.

## Data Availability

The datasets used and/or analysed during the current study available from the corresponding author on reasonable request.
